# Market Integration Predicts Human Gut Microbiome Attributes across a Gradient of Economic Development

**DOI:** 10.1128/mSystems.00122-17

**Published:** 2018-02-27

**Authors:** Keaton Stagaman, Tara J. Cepon-Robins, Melissa A. Liebert, Theresa E. Gildner, Samuel S. Urlacher, Felicia C. Madimenos, Karen Guillemin, J. Josh Snodgrass, Lawrence S. Sugiyama, Brendan J. M. Bohannan

**Affiliations:** a Institute of Ecology and Evolution, University of Oregon, Eugene, Oregon, USA; b Department of Anthropology, University of Colorado Colorado Springs, Colorado Springs, Colorado, USA; c Department of Anthropology, University of Oregon, Eugene, Oregon, USA; d Department of Anthropology, Hunter College (CUNY), New York City, New York, USA; e Department of Anthropology, Queens College (CUNY), New York City, New York, USA; f Institute of Molecular Biology, University of Oregon, Eugene, Oregon, USA; g Humans and the Microbiome Program, Canadian Institute for Advanced Research, Toronto, Ontario, Canada; University of Colorado Denver

**Keywords:** biological anthropology, market integration, microbial ecology, microbiome

## Abstract

Economic development is marked by dramatic increases in the incidence of microbiome-associated diseases, such as autoimmune diseases and metabolic syndromes, but the lifestyle changes that drive alterations in the human microbiome are not known. We measured market integration as a proxy for economically related lifestyle attributes, such as ownership of speciﬁc market goods that index degree of market integration and components of traditional and nontraditional (more modern) house structure and infrastructure, and proﬁled the fecal microbiomes of 213 participants from a contiguous, indigenous Ecuadorian population. Despite relatively modest differences in lifestyle across the population, greater economic development correlated with signiﬁcantly lower within-host diversity, higher between-host dissimilarity, and a decrease in the relative abundance of the bacterium *Prevotella*. These microbiome shifts were most strongly associated with more modern housing, followed by reduced ownership of traditional subsistence lifestyle-associated items.

**IMPORTANCE** Previous research has reported differences in the gut microbiome between populations residing in wealthy versus poorer countries, leading to the assertion that lifestyle changes associated with economic development promote changes in the gut microbiome that promote the proliferation of microbiome-associated diseases. However, a direct relationship between economic development and the gut microbiome has not previously been shown. We surveyed the gut microbiomes of a single indigenous population undergoing economic development and found signiﬁcant associations between features of the gut microbiome and lifestyle changes associated with economic development. These ﬁndings suggest that even the earliest stages of economic development can drive changes in the gut microbiome, which may provide a warning sign for the development of microbiome-associated diseases.

## Abstract (precis)

Previous research has reported differences in the gut microbiome between populations residing in wealthy versus poorer countries, leading to the assertion that lifestyle changes associated with economic development promote changes in the gut microbiome that promote the proliferation of microbiome-associated diseases. However, a direct relationship between economic development and the gut microbiome has not previously been shown. We surveyed the gut microbiomes of a single indigenous population undergoing economic development and found signiﬁcant associations between features of the gut microbiome and lifestyle changes associated with economic development. These ﬁndings suggest that even the earliest stages of economic development can drive changes in the gut microbiome, which may provide a warning sign for the development of microbiome-associated diseases.

## INTRODUCTION

It is increasingly evident that the gut microbiome—the collection of microbes found in the intestines of animals, including humans—plays a critical role in the development of various diseases, including metabolic syndrome and immunoallergic disease (1, 2). Previous studies suggest that people from wealthier nations (e.g., those in Western Europe and the United States) have gut microbiomes signiﬁcantly different from people from nations undergoing economic development (e.g., Africa, South America, and the Paciﬁc Islands) (3–8). This observation has led to the hypothesis that economic development results in substantial changes to the microbiome, resulting in the increased prevalence of major health problems associated with economic development, including cardiovascular disease, obesity, allergy, and autoimmune disorders (9–12). However, these assertions are derived from studies comparing the gut microbiomes of disparate populations (4–7), and thus confound the impact of economic development with other important factors that inﬂuence microbiome composition and diversity, such as ethnicity and geographic location (13, 14).

**FIG 1:**
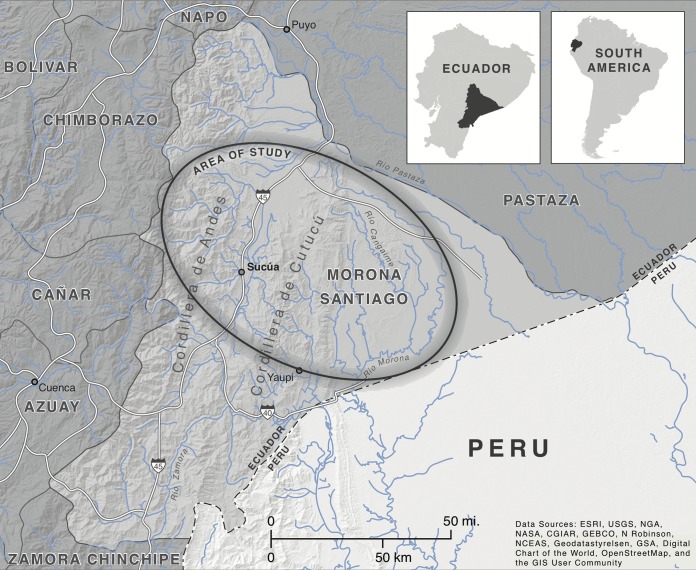
Map of Morona-Santiago province, Ecuador. The ellipse roughly corresponds to the area within which all ﬁve study villages reside. The two villages within the Upano Valley (west of the Cordillera de Cutucú and through which highway 45 runs), UV1 and UV2, have a travel time to the regional market center of Sucúa between 1 and 2 h (including a 30- to 60-min walk to the main road and a 30- to 60-min car or bus ride). Travel times to Sucúa from three villages east of the Cordillera de Cutucú vary between 7 and 12 h, based on the time of departure, weather conditions, and river height. Estimates for typical travel times from each Cross-Cutucú village are as follows: 8.5 to 9.5 h from CC1, 8 to 9 h from CC2, and 10.5 to 11.5 h from CC3. Josie Imrie created this figure for this paper, and it is used here with permission.

To test the role of economic development on intestinal microbiota diversity without such confounding factors, we conducted a survey of the fecal microbiome of a single indigenous population, the Shuar of southeastern Ecuador, and recorded household-level metrics of “market integration” (i.e., producing for and consuming from a market-based economy) to measure the level of economic development of the study participants (15–17). The Shuar are experiencing rapid market integration, but they share a recent common cultural and genetic history, having spread rapidly from a constrained geographic area in the last hundred years (Fig. 1). The degree of market integration varies between individuals, households, and communities but to a much lesser degree than between the populations studied in previous work. The impact of market integration on the health and well-being of the Shuar has been extensively studied (18–20). As a whole, the Shuar have favorable cardiovascular and metabolic health (e.g., analysis of C-reactive protein levels failed to ﬁnd a single “high-risk” case [21]), and market integration is associated with both positive and negative health outcomes (e.g., participants in the Upano Valley had higher high-density lipoprotein [HDL] levels, while those in Cross-Cutucú had higher diastolic blood pressure) (19, 20). However, little is known regarding how market integration inﬂuences the Shuar’s microbiomes.

For our study, samples were provided by participants living in ﬁve villages across a geographic region divided by the Cordillera de Cutucú mountain range in Ecuador (the number of participants per village can be found in Table S1 in the supplemental material). Two sample communities in the Upano Valley west of the Cordillera de Cutucú (UV1 and UV2) are approximately 1 h by truck from the town of Sucúa, a local market center. The Upano Valley is characterized by tropical low-montane forest, has an elevation of ~600 to 700 m, mean daytime temperatures of 24°C, and receives ~2,200-mm rainfall annually (22). Shuar in these communities tend to own more industrially produced items (e.g., televisions and portable propane stoves), and most reside in homes made from wood planks or recently introduced cinder block construction (19, 20). Three sample communities (CC1, CC2, and CC3) in the region east of the Cordillera de Cutucú mountain range (referred to as “Cross-Cutucú”) are much farther from market centers (1.5 to 3 h by motor canoe to a road where they might sell produce and an additional 5 to 8 h by bus to Sucúa). The Cross-Cutucú lies within the upper Amazonian flood plain with a lower elevation of ~200 to 300 m, mean daytime temperatures of 25°C, and ~3,000-mm annual rainfall (22). Residents of these villages tend to own more subsistence-associated items (e.g., hunting or ﬁshing equipment), more often live in traditional homes comprised of palm wood and thatch with dirt ﬂoors, and none live in cinderblock houses (19, 20). Climatic seasonality is mild in both regions.

10.1128/mSystems.00122-17.2 TABLE S1  Numbers of participants whose fecal samples passed quality controls in each village. Download TABLE S1, XLSX file, 0.02 MB. Copyright © 2018 Stagaman et al. 2018 Stagaman et al. This content is distributed under the terms of the Creative Commons Attribution 4.0 International license.

There is, nevertheless, substantial variation in market integration within each village, regardless of region (20). For example, some houses in the Upano Valley in Ecuador are still made using traditional materials, while more recently, houses in the Cross-Cutucú region have been built using wood planks. We therefore directly quantiﬁed the level of household market integration experienced by participants in this study, rather than simply using geographic location as a proxy measure of market integration, as previous studies have done (3–8). To do so, we used three lifestyle or style-of-life (SOL) metrics (see references 13 and 15 for details). The ﬁrst metric, SOL-House, is a composite metric of ﬁve codes indicating type of housing construction and infrastructure. The second metric, SOL-Traditional, is the proportion of important items owned that reﬂect investment in a traditional foraging lifestyle. The third, SOL-Market, is the proportion of important items owned that reﬂect degree of investment in manufactured goods associated with the market economy. The codes and items for these metrics can be found in Table S2.

10.1128/mSystems.00122-17.3 TABLE S2  Composite codes for the SOL-House metric and item lists for SOL-Traditional and SOL-Market metrics. Download TABLE S2, XLSX file, 0.04 MB. Copyright © 2018 Stagaman et al. 2018 Stagaman et al. This content is distributed under the terms of the Creative Commons Attribution 4.0 International license.

To reduce the number of variables in our analysis and to identify latent factors, we performed exploratory factor analysis, including all individual items used in the SOL metrics. The factor analysis produced three factors, which we call (in order of variance explained): house modernity, subsistence items, and power usage (the last indicating the number of objects owned that require external electrical or petrochemical power, such as radios, refrigerators, and gasoline engines). The results of the factor analysis and an explanation of the factor labels can be found in Table S3.

10.1128/mSystems.00122-17.4 TABLE S3  Results from factor analysis on the components of the SOL-House, SOL-Traditional, and SOL-Market metrics. The first factor is most strongly composed of the wall type and the floor type of a subject’s home, and to a lesser extent access to water and the type of latrine associated with the home. The more manufactured the materials used to build a subject’s house (e.g., cinder block versus palm wood), the higher their score for factor 1. Therefore, we named factor 1 “house modernity.” The second factor is almost exclusively defined by the proportion of objects a subject owns from the SOL-Traditional list, thus we called it “subsistence items.” The third factor’s strongest loadings are the level of access to electricity in a subject’s house and the proportion of objects a subject owns from the SOL-Market list, which is mostly composed of items that use either electrical or petrochemical power. Factor 3 is therefore called “power usage.” Download TABLE S3, XLSX file, 0.04 MB. Copyright © 2018 Stagaman et al. 2018 Stagaman et al. This content is distributed under the terms of the Creative Commons Attribution 4.0 International license.

## RESULTS

On the basis of previous studies suggesting that market integration is inversely related to intraindividual microbiome diversity (α-diversity) (3–8), we predicted a negative correlation between the phylogenetic diversity (PD) of the gut microbiome and the factors associated with greater market integration, the house modernity and power usage factors. Similarly, we expected a positive correlation between PD and the subsistence item factor. As detailed in Materials and Methods, we performed model selection starting from a full model that included all three style-of-life factors, participant age, and the rank travel time from Sucúa, Ecuador, and determined that the best-ﬁt model included only age, region (Upano Valley in Ecuador versus Cross-Cutucú in Ecuador), house modernity, and power usage.

**FIG 2:**
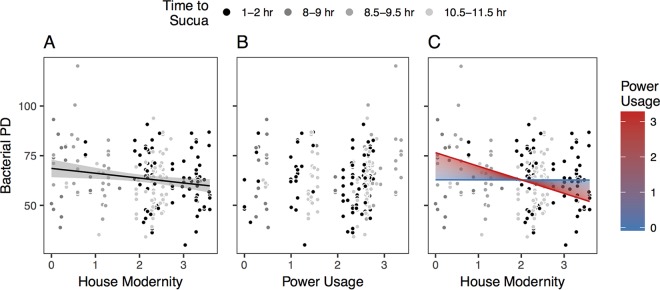
Phylogenetic diversity (PD) by signiﬁcant market integration factors, house modernity (A), power usage (B), and house modernity and power usage (C). (A) House modernity (factor 1). The black line is the best-ﬁt line from regressing PD by house modernity (*R*^*2*^ = 0.024; *P* = 0.013). (B) Power usage (factor 3) (not statistically significant). (C) Interaction between house modernity and power usage (*R*^*2*^ = 0.037; *P* = 0.012). The blue line is the predicted relationship (using the full regression model) between PD and house modernity when power usage is held at zero. The red line is the predicted relationship when power usage is set at its maximum, and the gradient between the two prediction lines represents predictions for each of 100 steps between the minimum and maximum values of power usage. *n =* 213 for all panels.

Because age followed the expected trends and did not interact with any other factors (see Table S4 in the supplemental material), we omitted it from the rest of the analyses. Figure 2A shows the predicted signiﬁcant negative relationship between PD and house modernity. That is, participants with homes built from more modern materials have lower gut microbiome phylogenetic diversity than people with homes built from more traditional materials. While there was a significant main effect of region, there was no interaction with the SOL factors. However, we included region in the subsequent models to determine whether the SOL factors explained variance in diversity beyond what could be explained by region alone (Fig. 2).

10.1128/mSystems.00122-17.5 TABLE S4  Significance of terms in the full model for predicting phylogenetic diversity (PD; *n* = 213). Terms with *P* values of less than 0.05 are shown in boldface type. Download TABLE S4, XLSX file, 0.04 MB. Copyright © 2018 Stagaman et al. 2018 Stagaman et al. This content is distributed under the terms of the Creative Commons Attribution 4.0 International license.

There was no significant relationship between PD and subsistence items or power usage (Fig. 2B). However, there was a signiﬁcant interaction between power usage and house modernity such that as the power usage of participants increases, the strength of relationship between PD and house modernity increases (Fig. 2C). Thus, house modernity and power usage appear to be separate, but related, measures of market integration that are signiﬁcantly associated with the diversity of the human gut microbiome.

**FIG 3:**
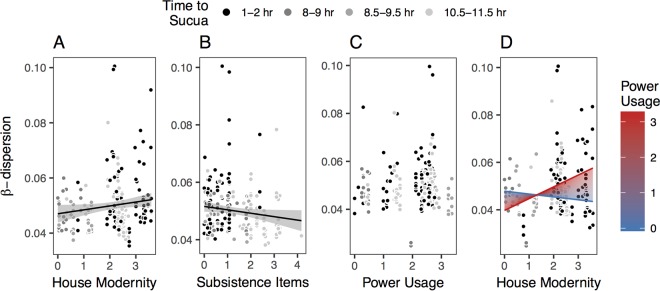
β-Dispersion by each market integration factor. The term β-dispersion is often used when comparing the β-diversity of subjects within the same treatment or group. (A) House modernity (*n* = 212; *R*^*2*^ = 0.014; *P* = 0.045). (B) Subsistence items (*n* = 213; *R*^*2*^ = 0.014; *P* = 0.046). (C) Power usage (*n* = 213) (not statistically significant). (D) Interaction between house modernity and power usage (*n* = 209; *R*^*2*^ = 0.034, *P* = 0.018). β-Dispersion was calculated as described in Materials and Methods. The black lines represent the best-ﬁt regression lines for β-dispersion by each individual factor. The colored lines in panel D represent the predicted relationship between β-dispersion and house modernity when power usage is held at zero up to its maximum observed value, divided into 100 steps.

Previous studies that compared disparate populations found that those in regions with higher market integration tend to have greater among-subject variation (β-diversity) than more traditionally living populations (6, 7). It is hypothesized that this may be due to either lower levels of exposure to a common pool of environmental microbes or lower levels of microbial dispersal between individuals (6). We predicted that greater house modernity and power usage would be associated with greater dissimilarity among participants’ microbiomes, whereas higher subsistence item scores would be associated with greater homogeneity of participants’ microbiomes. We calculated the mean weighted UniFrac (23) distance between the gut microbiomes of each subject and those of other subjects who experience similar levels of market integration (see Materials and Methods for details). These analyses conﬁrmed our hypotheses: house modernity was positively associated with among-subject variation (i.e., microbiomes were more dissimilar as house modernity increased; Fig. 3A), while subsistence items were negatively related to among-subject variation (i.e., microbiomes were more homogeneous as subsistence items increased; Fig. 3B). Alone, power usage did not have a signiﬁcant effect on among-subject variation (Fig. 3C). However, as with within-host diversity, there was a signiﬁcant interaction between house modernity and power usage (Fig. 3D), such that as power usage increases, the strength of the relationship between house modernity and among-subject variation increases.

**FIG 4:**
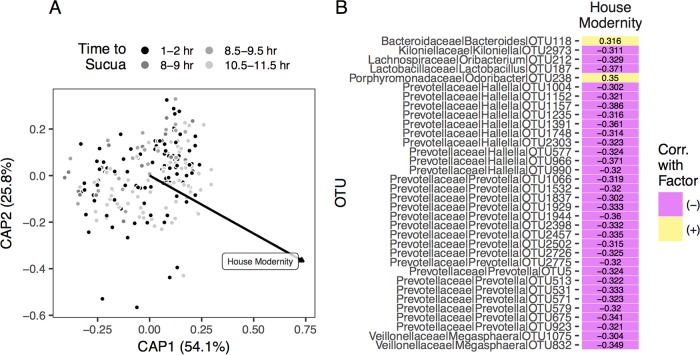
Intestinal microbiota composition. (A) Distance-based RDA ordination of bacterial community distances overlaid with signiﬁcant market integration factor vector, house modernity (*n* = 213; *P* = 0.008). CAP in the axes stands for constrained analysis of principal components and is an alternative term for distance-based redundancy analysis. (B) Statistically signiﬁcant correlation coefficients for OTU abundances versus house modernity, organized alphabetically by taxonomic family. Positive and negative correlations are shown.

We analyzed the taxonomic composition of the gut microbiome of each subject via distance-based redundancy analysis (db-RDA) (Fig. 4A) and permutational analysis of variance (PERMANOVA) (Table S5). These analyses reveal that house modernity is signiﬁcantly associated with gut microbiome composition. We included participant region in the db-RDA analysis as a “Condition” variable, which means that its variance is “partialled out” by the analysis before considering the significance of the other variables.

10.1128/mSystems.00122-17.6 TABLE S5  Result of PERMANOVA analysis of contribution of style-of-life factors to microbiota composition (*n* = 213). Terms with *P* values of less than 0.05 are shown in boldface type. Download TABLE S5, XLSX file, 0.04 MB. Copyright © 2018 Stagaman et al. 2018 Stagaman et al. This content is distributed under the terms of the Creative Commons Attribution 4.0 International license.

Finally, a multiple correlation test (*α* = 0.05, false-discovery rate corrected) of the relationships among the abundances of all microbiome taxa and the three market integration factors revealed 32 operational taxonomic units (OTUs) that were negatively correlated with house modernity and two OTUs that were positively correlated with house modernity (Fig. 4B). Of these 32 OTUs, 16 were assigned to the genus *Prevotella*, and another 10 were assigned to the genus *Hallela*, a member of the *Prevotellaceae* family. Of the two OTUs positively correlated with house modernity, one was assigned to *Bacteroides*. These results are consistent with previous studies (3, 4, 7). For example, Yatsunenko et al. (4) reported that 23 of 73 OTUs that were overrepresented in Amerindian or Malawian versus U.S. adults were assigned to *Prevotella*, and De Filippo et al. (3) found that the intestinal microbiomes of participants from Burkina Faso harbored a much larger proportion of *Prevotella* than those of participants from the United States. Additionally, Yatsunenko et al. (4) reported a negative relationship between the abundance of *Prevotella* and *Bacteroides* in adults, while De Filippo et al. (3) reported a greater proportion of *Bacteroides* in microbiomes from U.S. individuals relative to microbiomes from Burkina Faso individuals.

## DISCUSSION

Our results suggest that even within a single ethnicity living in a constrained geographic region, the early stages of market integration affect the diversity and composition of the gut microbiome. In particular, the modernity of participants’ homes consistently predicts gut microbiome attributes. The mechanism by which house modernity affects the gut microbiome cannot be deﬁnitively determined from our study, but it could plausibly be due to the isolation from environmental microbes afforded by more modern housing. For example, related work with the Shuar showed reduced exposure to helminth soil parasites in more modern homes (24). Traditional housing consists of palm thatch structures with dirt ﬂoors, which allow more exposure to microbes from the “outside” (i.e., those associated with soil and plants) than does more modern housing (which consists of wood or cinder block structures with plank or concrete ﬂoors). The idea that more modern housing excludes environmental microbes is consistent with our previous work associating house modernity with reduced exposure to soil-transmitted parasites (24), as well as previous work by other researchers showing that more modern housing does indeed exclude environmental microbes from the built environment (25). The intensifying effect of power usage on the relationship between house modernity and microbiome diversity metrics may be the result of numerous lifestyle changes that reduce a person’s exposure to environmental microbes, such as remaining in their homes to use powered devices, employment in jobs (such as teaching) that are primarily indoors, or having access to a vehicle and a refrigerator increases the likelihood that food is bought commercially rather than foraged, ﬁshed, or hunted. Ownership of subsistence items, on the other hand, could be positively correlated with environmental microbe exposure associated with outdoor activities and nondomesticated animals, such as hunting. Alternatively, subsistence items and house modernity (and its interaction with power usage) may together be a proxy for a suite of other lifestyle factors (e.g., dietary changes, health care practices, etc.) associated with economic development, which could be the actual drivers of the microbiome differences we observed.

Cardiovascular disease is now the leading cause of death in all nations but those with the lowest incomes (9). Obesity, already a major public health problem in wealthier nations, is rapidly increasing in the developing world (9). Allergy and autoimmune disorders continue to rise in the west (11). The increasing incidence of these and other microbiome-associated disorders currently experienced by populations in wealthy nations has been hypothesized to be driven by the loss of microbes essential to human health (the “hygiene hypothesis” [26] and the “disappearing microbiota hypothesis” [27]). These hypotheses assert that recent lifestyle changes have either limited our exposure to or have driven extinct certain members of the microbiome in economically developed nations. The association between early market integration and gut microbiome composition and diversity observed in our study demonstrates that economic development can, indeed, alter the human microbiome, as predicted by these hypotheses. Furthermore, we show that these changes occur even in the early stages of market integration. Our results are consistent with the assertion that reduced exposure to environmental microbes is a driver of microbiome changes in economically developing countries, although further research is needed to deﬁnitively test this hypothesis. Finally, our results suggest that the microbiome differences we observed may provide an early warning sign for microbiome-associated disorders in rapidly developing countries. That is, while there are no strong indications of decreasing health or well-being in the participating populations, their microbiomes exhibit changes observed in more economically developed countries where microbiome-associated diseases are prevalent.

## MATERIALS AND METHODS

### Quantiﬁcation of market integration and factor analysis.

The three style-of-life (SOL) metrics were determined as described in previous work (19, 20). In short, researchers conducted structured interviews, administered mostly in Spanish (or through a bilingual translator for subjects who did not speak Spanish), to collect a range of demographic and lifestyle information. The ages of the participants ranged from 1 to 100 years. Dietary data were collected in the form of a food frequency questionnaire. However, as we did not directly quantify caloric amount and nutritional content of food consumed by each participant, and we had diet data for only 140 of the 213 participants for whom we have microbiome data, diet data were excluded from the primary analysis. Analysis of the diet data we do have produced no signiﬁcant associations between bacterial PD (see Table S6 in the supplemental material) or composition (Table S7). The lack of significance for either diet or SOL factors in these models is most likely due to the reduction in power of reducing our sample size by 73 samples (a reduction of ~35%). Ethnographic observations and pilot testing over the course of a decade led to the selection of items in the house, traditional, and market style-of-life metrics. The ﬁnal SOL-Traditional scale contained six items reﬂecting investment in a foraging lifestyle, while the SOL-Market scale included 12 items reﬂecting investment in a market economy. Individual scores were calculated as the fraction of list items owned (range, 0 to 1). The SOL-House metric included ﬁve household measures as indices of household permanence, access to infrastructure, market participation, and pathogen risk. We conducted an exploratory factor analysis on the two item-based metrics (SOL-Traditional and SOL-Market), along with the ﬁve components of the SOL-House metric (type or presence of wall, ﬂoor, bathroom, water, and electricity in a participant’s home) using the factanal function from the basic R stats package (28). Starting with ﬁtting a single factor, we increased the number of ﬁtted factors until either we reached the maximum allowed by the method (three for seven input variables) or until the *P* value of the analysis was less than 0.05. This analysis resulted in three market integration factors that were similar to the style-of-life metrics except that the electricity type (from SOL-House) loaded most strongly on the third factor with SOL-Market. Biplots from the factor analysis can be found in Fig. S1, and all associated metadata can be found in Table S8.

10.1128/mSystems.00122-17.7 TABLE S6  Result of linear regression analysis of style-of-life factors, diet factors, and bacterial phylogenetic diversity (PD) (*n* = 140). Terms with *P* values of less than 0.05 are shown in boldface type. Download TABLE S6, XLSX file, 0.04 MB. Copyright © 2018 Stagaman et al. 2018 Stagaman et al. This content is distributed under the terms of the Creative Commons Attribution 4.0 International license.

10.1128/mSystems.00122-17.8 TABLE S7  Results of PERMANOVA analysis of contribution of style-of-life and diet factors to microbiota composition (*n* = 140). Terms with *P* values of less than 0.05 are shown in boldface type. Download TABLE S7, XLSX file, 0.04 MB. Copyright © 2018 Stagaman et al. 2018 Stagaman et al. This content is distributed under the terms of the Creative Commons Attribution 4.0 International license.

10.1128/mSystems.00122-17.9 TABLE S8  Metadata for all fecal samples. Download TABLE S8, XLSX file, 0.04 MB. Copyright © 2018 Stagaman et al. 2018 Stagaman et al. This content is distributed under the terms of the Creative Commons Attribution 4.0 International license.

10.1128/mSystems.00122-17.1 FIG S1  Biplots of item codes and style-of-life metrics with factor scores for each participant. The contribution of item codes and style-of-life metrics to each factor are represented by the direction and magnitude of its labeled green vector. Points represent scores for each participant (*n* = 213) for each factor and are colored by the average travel time from each village to Sucúa, Ecuador. Ellipses represent the standard error around the centroid for each estimated travel time. The top panel plots house modernity versus subsistence item scores. The bottom panel plots house modernity versus power usage scores. Download FIG S1, PDF file, 0.02 MB. Copyright © 2018 Stagaman et al. 2018 Stagaman et al. This content is distributed under the terms of the Creative Commons Attribution 4.0 International license.

### Stool collection and DNA extraction.

Three hundred stool samples were collected as described previously (18). Brieﬂy, participants were given a prepacked plastic bag containing an empty stool container and clean implements with which to collect the stool sample and instructed on the collection technique. Participants returned the containers, and samples were preserved in RNAlater (Thermo Fisher Scientiﬁc, Waltham, MA, USA) within an hour of sample collection. Preserved samples were stored in a portable freezer at −20°C over the course of data collection and then shipped to the lab on dry ice, where it was stored at −80°C until analysis. DNA was extracted from the samples using the blood and stool kit (Qiagen, Hilden, Germany) in accordance with the kit protocol. No human genetic data were gathered as part of this project, and the bacterial data gathered were purged of all sequences that aligned to the human genome (including mitochondrial genome) before archiving. Genetic material resulting from this research will never be used for human DNA research or commercial cell line patenting.

### Ethics statement.

Informed verbal consent was obtained from adult participants. For participants under 15 years old (the local age of consent), parental verbal consent and child assent were obtained. Individuals were informed that they could choose not to participate, to participate only in individual portions of the study, or to participate in the full study. The study and consent procedures were approved by the Institutional Review Board (IRB) of the University of Oregon, and a central Shuar governing organization authorized research in member villages. The precise locations of the villages in Ecuador were omitted from Fig. 1 to protect the anonymity of the participants.

### Illumina library preparation and 16S rRNA gene sequence analysis.

We characterized the intestinal microbial communities of fecal samples via Illumina (San Diego, CA) sequencing of 16S rRNA gene amplicons. To prepare amplicons for Illumina sequencing, we used a single-step PCR method to add dual indices and adapter sequences to the V4 region of the bacterial 16S rRNA gene (no human sequences were speciﬁcally targeted) and generate paired-end 150-nucleotide reads on the Illumina HiSeq 2000 platform.

The 16S rRNA gene Illumina reads were processed using methods implemented by FLASH (29), the FASTX Toolkit (http://hannonlab.cshl.edu/fastx_toolkit/), and the USEARCH pipeline (30). The processing pipeline can be found at http://www.github.com/kstagaman/Process_16S. Operational taxonomic units (OTUs) were deﬁned using 97% sequence similarity. Any amplicons that matched the human genome were removed from the analysis with bowtie (31) prior to OTU clustering. Read assembly, quality control, and OTU table building were done on the University of Oregon ACISS cluster, and all subsequent data processing and diversity analyses were done in R (28).

### Intestinal microbiota diversity analyses.

Samples were not included in the analysis if they had fewer than 20,000 total reads or were from individuals lacking complete SOL metric data. After quality control, the distribution of sequences per samples was 20,843 to 2,610,907 (median, 168,951). OTU abundances of the remaining 213 samples were variance stabilized using phyloseq (32) and DESeq2 (33) as recommended (34). (A parallel analysis was conducted by rarefying all samples to 20,843 sequences and did not change the interpretation of the results.) We measured phylogenetic diversity using Faith’s PD (35), which takes into account taxon abundances as well as their phylogenetic relationship, as implemented in the picante package (36), and chose the best linear model using the anova function from the base R stats package (28). We used the distance function from the phyloseq package to calculate weighted UniFrac distances (23) between microbiomes. When comparing the β-diversity of subjects within the same treatment or group, the term β-dispersion is often used. We calculated β-dispersion as the mean weighted UniFrac community distance between each participant and other participants within 5% of the same factor score (thus comparing similarly market-integrated participants; analyses using between 2.5 and 10% of factor scores resulted in qualitatively similar results). Using the same distance matrix, we generated a distance-based redundancy analysis (db-RDA) ordination using the cap scale function and measured individual factor R-squared values via permutational analysis of variance (PERMANOVA) using the adonis function, both from the vegan package (37). Other distance metrics were used and produced qualitatively similar results. We conducted a multiple correlation test on the OTU table and the market integration factors using the corr.test function from the psych package (38), which uses the base cor function to ﬁnd correlations and then applies a *t* test to the individual correlations using the formula. The function then applies a correction to the *P* values using the base function p.adjust, for which we chose the “BY” variant of the false-discovery rate (39). Diversity data visualization was done with the ggplot2 (40), ggfortify (41), and ggbiplot (42) packages.

### Data availability.

Sequences were deposited under BioProject accession number PRJNA362944.
