# Supplementary figures and images for: Market Integration Predicts Human Gut Microbiome Attributes across a Gradient of Economic Development
Source: mSystems. 2018 Feb 27;3(1):e00122-17. doi: 10.1128/mSystems.00122-17 (PMC5829308; doi:10.1128/mSystems.00122-17)

A

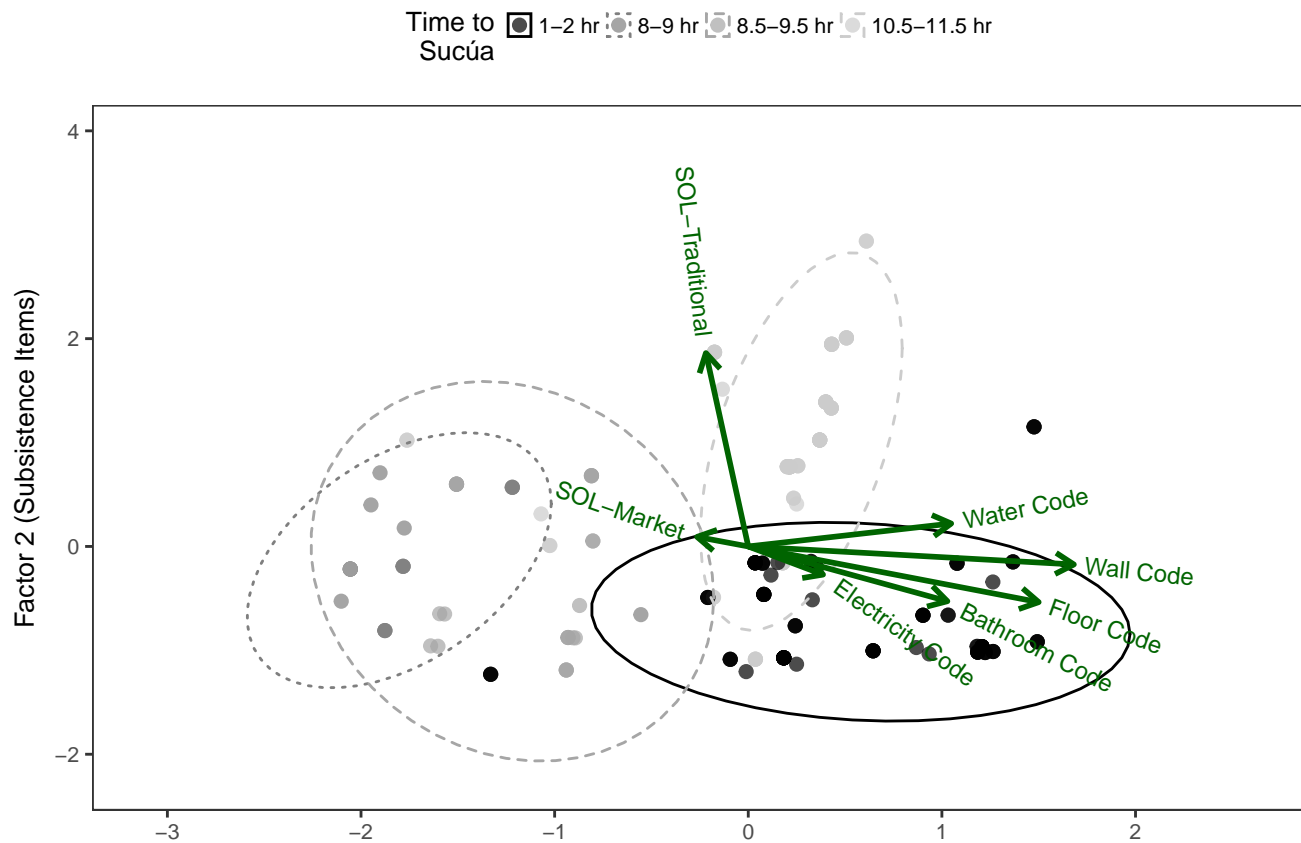

B

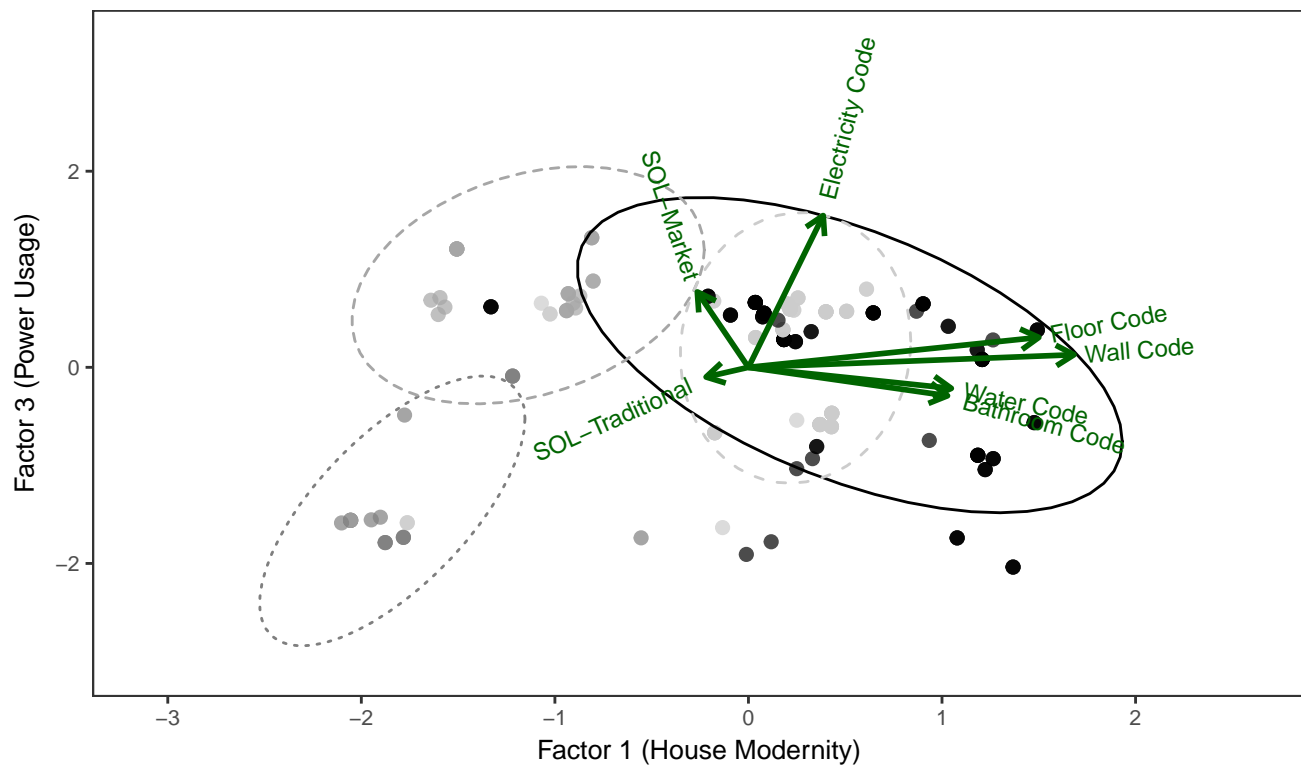

Supplement: FIG S1 [file sys001182195sf1.pdf]
